# MFPI-Net: A Multi-Scale Feature Perception and Interaction Network for Semantic Segmentation of Urban Remote Sensing Images

**DOI:** 10.3390/s25154660

**Published:** 2025-07-27

**Authors:** Xiaofei Song, Mingju Chen, Jie Rao, Yangming Luo, Zhihao Lin, Xingyue Zhang, Senyuan Li, Xiao Hu

**Affiliations:** 1 School of Automation and Information Engineering, Sichuan University of Science and Engineering, Yibin 644005, China; 323085404418@stu.suse.edu.cn (X.S.); 323085404110@stu.suse.edu.cn (J.R.); 324085404516@stu.suse.edu.cn (Y.L.); 324085404512@stu.suse.edu.cn (Z.L.); 323081104117@stu.suse.edu.cn (X.Z.); 323085404108@stu.suse.edu.cn (S.L.); 323085404103@stu.suse.edu.cn (X.H.); 2 Intelligent Perception and Control Key Laboratory of Sichuan Province, Sichuan University of Science and Engineering, Yibin 644005, China

**Keywords:** urban remote sensing imagery, semantic segmentation, multi-scale, contextual information, feature fusion

## Abstract

To improve semantic segmentation performance for complex urban remote sensing images with multi-scale object distribution, class similarity, and small object omission, this paper proposes MFPI-Net, an encoder–decoder-based semantic segmentation network. It includes four core modules: a Swin Transformer backbone encoder, a diverse dilation rates attention shuffle decoder (DDRASD), a multi-scale convolutional feature enhancement module (MCFEM), and a cross-path residual fusion module (CPRFM). The Swin Transformer efficiently extracts multi-level global semantic features through its hierarchical structure and window attention mechanism. The DDRASD’s diverse dilation rates attention (DDRA) block combines convolutions with diverse dilation rates and channel-coordinate attention to enhance multi-scale contextual awareness, while Shuffle Block improves resolution via pixel rearrangement and avoids checkerboard artifacts. The MCFEM enhances local feature modeling through parallel multi-kernel convolutions, forming a complementary relationship with the Swin Transformer’s global perception capability. The CPRFM employs multi-branch convolutions and a residual multiplication–addition fusion mechanism to enhance interactions among multi-source features, thereby improving the recognition of small objects and similar categories. Experiments on the ISPRS Vaihingen and Potsdam datasets show that MFPI-Net outperforms mainstream methods, achieving 82.57% and 88.49% mIoU, validating its superior segmentation performance in urban remote sensing.

## 1. Introduction

Image semantic segmentation is an important research direction in the field of computer vision. Its purpose is to assign each pixel in the input image to the corresponding semantic category, so as to achieve the accurate division of different land object areas. This technology is widely used in unmanned driving, intelligent medical care, geographic information systems, and other fields. Especially in the field of remote sensing image processing, semantic segmentation technology is of great significance to tasks such as urban planning [1,2], land use monitoring [3,4], environmental assessment [5], and disaster response [6]. Compared with natural images, the semantic segmentation of urban remote sensing images faces more severe challenges: On the one hand, there are many types of objects in the image and the scale changes dramatically. There are both large-scale building complexes and small targets that are difficult to distinguish (such as vehicles and occlusions) [7], which puts high demands on the multi-scale modeling ability of the model. On the other hand, there is a high degree of texture similarity between objects (such as roofs and roads, bare land, and buildings). In addition, remote sensing images have a wide coverage area and complex spatial relationships. It is impossible to fully model global context information by relying solely on local perception, especially in complex urban terrain and heterogeneous coverage scenes. The difficulty of semantic understanding is significantly increased.

In recent years, deep learning methods have developed rapidly and achieved remarkable results in semantic segmentation tasks. However, existing methods still face many challenges in processing urban remote sensing images. Although traditional convolutional neural networks (CNNs) perform well in extracting local features [8], they have difficulty modeling long-distance dependencies [9]. Transformer-based methods have excellent global modeling capabilities [10], but they have high computational overhead when processing high-resolution images and are weak in capturing local details [11]. Although some current hybrid architectures have integrated the advantages of both to a certain extent [12], they still have shortcomings in multi-scale target recognition, high similarity between categories, and small target detection.

In general, although deep learning technology has promoted the rapid development of semantic segmentation, urban remote sensing image segmentation still faces many challenges, such as multi-scale object distribution, strong category similarity, and small targets are easily missed. Especially in the case of dense targets, blurred boundaries, and complex scenes, the existing models have limited capabilities in global semantic modeling and context understanding, and it is difficult to effectively capture fine-grained structures and accurately distinguish similar categories. These problems have largely restricted the actual performance of the model in urban remote sensing semantic segmentation tasks.

In order to meet these challenges and further improve the accuracy of remote sensing image semantic segmentation, this paper proposes an encoder–decoder structure based on multi-scale feature perception and interaction for urban remote sensing image semantic segmentation network MFPI-Net. It consists of four main parts: an encoder with Swin Transformer as the backbone, a diverse dilation rates attention shuffle decoder (DDRASD) composed of a diverse dilation rates attention block (DDRA Block) and a Shuffle Block, a multi-scale convolutional feature enhancement module (MCFEM), and a cross-path residual fusion module (CPRFM). Although there are many hybrid models that combine Transformer, attention mechanism, and multi-scale fusion, the existing methods still have shortcomings in the effective fusion of multi-scale information and information flow design. MFPI-Net uses innovative multi-scale fusion strategies and specially designed attention decoders to achieve more complete feature interaction and information integration, thereby improving the model’s expressiveness and performance. Our research contributions are summarized as follows:

- We adopt the Swin Transformer as the encoder backbone [13], leveraging its hierarchical structure and shifted window self-attention mechanism to effectively model spatial dependencies and contextual relationships of complex targets in urban remote sensing images, while maintaining high computational efficiency. The Swin Transformer extracts and compresses image features layer by layer, generates multi-level, globally consistent semantic representations, and provides strong expressive feature support for subsequent modules, thereby significantly improving the model’s structural modeling capabilities and segmentation performance in complex remote sensing scenes.
- We designed the DDRASD consisting of the DDRA Block and the Shuffle Block to efficiently decode semantic information from multi-scale feature maps. Specifically, the DDRA Block obtains information from multiple scales and a wide range of contexts for decoding through different dilation rates, while introducing channel and coordinate attention mechanisms to enhance feature representation and improve the model’s ability to detect and segment objects of different sizes. The Shuffle Block achieves resolution improvement by rearranging the pixels of the input feature map to avoid the checkerboard artifacts that may be caused by traditional deconvolution.
- We conceived the CNN-based MCFEM to enhance the modeling capability of multi-scale targets in remote sensing images. This module constructs parallel branches through different convolution kernels to perceive spatial context information from fine-grained to large-scale. The MCFEM is integrated into the network input stage and the jump connection path of the encoder, introducing local spatial structure and multi-scale details, effectively making up for the shortcomings of Swin Transformer in edge details and local texture modeling, achieving efficient fusion of spatial details and global semantic information, and improving overall segmentation performance.
- We designed the CPRFM to efficiently integrate feature information from different network layers. We constructed a multi-branch feature path through global context, local convolution, and point convolution to extract multi-scale information. In the fusion stage, we adopted a feature-enhanced residual multiplication and addition mechanism to effectively strengthen the complementary relationship and distinguishing ability between different features, achieve more delicate feature interaction and information integration, and effectively improve the recognition ability of subtle targets and similar categories in complex environments.

## 2. Related Work

With the rapid development of deep learning technology, semantic segmentation methods have undergone a transformation from early traditional algorithms to deep learning methods. The current mainstream semantic segmentation methods mainly include local feature extraction methods based on CNN [14] and global context modeling methods based on Transformer [15]. In recent years, researchers have gradually explored hybrid architectures [16] that combine the advantages of the two, by introducing convolutional modules to enhance the Transformer’s modeling capabilities for local details, or embedding self-attention mechanisms in convolutional networks to enhance global perception, effectively taking into account both fine structure expression and semantic modeling capabilities. On this basis, in order to further enhance the model’s detail perception and multi-scale fusion capabilities, researchers have successively proposed a variety of structural optimization strategies and enhancement modules [17,18,19,20].

### 2.1. Semantic Segmentation Methods

Semantic segmentation methods based on deep learning can be mainly divided into two categories: one is based on CNN, focusing on the extraction and modeling of local spatial features; and the other is based on the Transformer architecture, which enhances the understanding of the global semantic relationship of the image through the self-attention mechanism. Early methods were mainly based on CNN. The Fully Convolutional Network (FCN) [21] proposed by Long et al. replaced the fully connected layer with the convolutional layer for the first time to achieve end-to-end pixel-level prediction. Although its structure is simple and efficient, laying the foundation for semantic segmentation, it has shortcomings in multi-scale target recognition and boundary recovery, especially in the weak recognition of small targets in remote sensing images. Subsequently, Zhao et al. proposed PSPNet [22], adding a pyramid pooling module (PPM) after the feature extraction network to improve the scene parsing ability through multi-scale context information, but it still has limitations in small target detection and edge detail processing. In order to further expand the receptive field, Chen et al. proposed the DeepLab series [23], such as DeepLabV3+ [24], which introduced the dilated convolution and dilated spatial pyramid pooling (ASPP) structure to enhance the context expression ability while maintaining resolution. However, dilated convolution at high dilation rates may lead to sparse sampling, which may cause a checkerboard effect and affect the modeling of boundaries and small objects. The U-Net [25] proposed by Ronneberger et al. uses a symmetrical encoding–decoding structure and fuses shallow spatial details with deep semantic features through skip connections, which performs well in medical images and remote sensing images. However, since its essence is still based on local convolution, it is difficult to model long-distance dependencies [26].

With the success of Transformer in natural language processing [27], its powerful global modeling ability has been introduced into the field of computer vision. Dosovitskiy et al. proposed Vision Transformer (ViT) [28], which captures the semantic relationship between arbitrary positions through the self-attention mechanism and demonstrates excellent global modeling ability. However, ViT has high computational overhead when processing high-resolution images and lacks the ability to depict local details, which limits its direct application in remote sensing images. In order to balance global modeling ability and computational efficiency, Liu et al. proposed Swin Transformer [29], which adopts a hierarchical structure and shifted window self-attention mechanism to expand the receptive field while controlling the amount of computation. It has now become one of the mainstream backbone networks in remote sensing semantic segmentation. However, its local window mechanism may cause context fragmentation when processing cross-region or semantically similar regions, affecting global consistency modeling. In order to enhance the feature parsing ability of Swin Transformer in downstream semantic segmentation tasks, Cao et al. proposed Swin-UNet [30], which introduced Swin Transformer into the U-Net framework, fused multiple layers of features through skip connections, and improved the model’s ability to parse complex structures. However, category confusion may still occur in highly similar regions and scenes with dense details. Similarly, Chen et al. proposed TransUNet [31], which introduced the Transformer module in the encoder to enhance the global modeling capability and retain the strong representation ability of CNN, achieving an effective combination of structural and semantic information in medical images, providing inspiration for remote sensing semantic segmentation. In addition, Cheng et al. proposed Mask2Former [32], which unified semantic segmentation into a mask prediction problem, introduced a query-based segmentation mechanism and a multi-scale decoder, significantly improving the versatility and performance of the model in multi-class segmentation tasks, and also demonstrated the potential of the Transformer architecture in a unified modeling framework.

In order to make up for the limitations of CNN and Transformer, a variety of hybrid architectures [33,34,35] that combine the advantages of both have emerged in recent years. Wang et al. proposed DC-Swin [36], which combines the hierarchical modeling capabilities of Swin Transformer with the multi-scale feature fusion strategy, effectively improving the semantic segmentation performance in complex remote sensing scenes; Zhou et al. proposed EAS-CNN [37], which optimizes the convolutional network structure based on the neural architecture search method, adaptively constructs semantic representation, and enhances the generalization ability of the model.

### 2.2. Structural Optimization Strategy and Feature Enhancement Module

In view of the complex and changeable characteristics of remote sensing images, designing effective structural optimization strategies and feature enhancement modules is crucial to improving the performance of semantic segmentation models. These methods mainly focus on enhancing multi-scale feature expression, improving detail recovery capabilities, and achieving deep fusion of contextual information.

In terms of structural optimization, HRNet [38] proposed by Sun et al. always retains high-resolution features through multi-resolution parallel branches, significantly improving the ability of boundary restoration and small target recognition. However, its complex structure and high consumption of computing resources limit its application in real-time remote sensing scenarios. In the decoding stage, Shi et al. introduced the Pixel Shuffle [39] operation to reconstruct high-resolution feature maps by rearranging channels, effectively alleviating the common checkerboard artifact problem of deconvolution. Some studies have proposed a hybrid upsampling strategy that combines bilinear interpolation and convolution to balance detail retention and computational efficiency. In addition, the residual attention-guided fusion strategy can effectively balance the semantic information between features at different levels, further improving the segmentation effect of boundaries and complex structure areas.

In terms of feature enhancement, the attention mechanism shows excellent performance. The Squeeze-and-Excitation (SE) module [40] proposed by Hu et al. recalibrates feature expressions through the channel attention mechanism; the Convolutional Block Attention Module (CBAM) [41] designed by Woo et al. combines channel and spatial attention to further focus on key area information; the coordinate attention module [42] proposed by Hou et al. introduces position information in attention modeling, enhancing the model’s adaptability to complex spatial structures.

### 2.3. Deficiencies of Existing Methods and Research Motivation

Although a large number of studies in recent years have attempted to combine Transformer with strategies such as multi-scale modeling and attention mechanisms to improve the model’s ability to understand complex visual tasks, current methods still have obvious deficiencies in feature interaction, fusion methods, and decoding strategies. First, many methods focus on sequential stacking at the module level and lack cross-layer or cross-path feature interaction mechanisms, resulting in loose connections between local and global information and difficulty in building a unified and continuous semantic representation. Secondly, mainstream feature fusion strategies mostly rely on static splicing or linear weighting, ignoring the differences in semantic consistency and spatial distribution of multi-source features, and are therefore prone to information confusion when dealing with small targets, complex backgrounds, or highly similar categories. In addition, in the decoding stage, existing methods generally use fixed-structure upsampling modules, and the attention mechanism is mostly limited to encoders or shallow feature processing, lacking deep fusion and flexible application in the decoding process, making it difficult to balance the restoration quality of high-resolution features and the accurate recovery of contextual information, and affecting the final prediction performance.

To solve the above problems, this study proposed MFPI-Net. Its core innovation is not to simply stack new modules, but to build a collaborative framework that integrates multi-scale feature enhancement, cross-path interaction fusion, and context-aware decoding through structural reconstruction and information flow optimization. MFPI-Net emphasizes dynamic integration in spatial and semantic dimensions, strengthens semantic complementarity and spatial alignment between multi-source features, and thus significantly improves the modeling ability of complex structures and multi-scale targets while maintaining computational efficiency. Especially in the decoding stage, MFPI-Net innovatively integrates the attention mechanism deeply into the multi-scale context modeling and spatial rearrangement modules to build a decoder with a flexible structure and strong context awareness. Unlike traditional methods that limit attention to encoders or shallow features, the attention mechanism in the MFPI-Net decoder can dynamically adjust the importance of multi-scale features, achieving precise attention to salient areas and detail enhancement, which not only effectively improves the expression and restoration accuracy of high-resolution features, but also significantly suppresses artifacts and blurring. Through this design, the network achieves a better balance between detail preservation and global understanding, filling the gap in existing methods in feature interaction, fusion, and deep integration of decoding mechanisms.

## 3. Methodology

**Figure 1:**
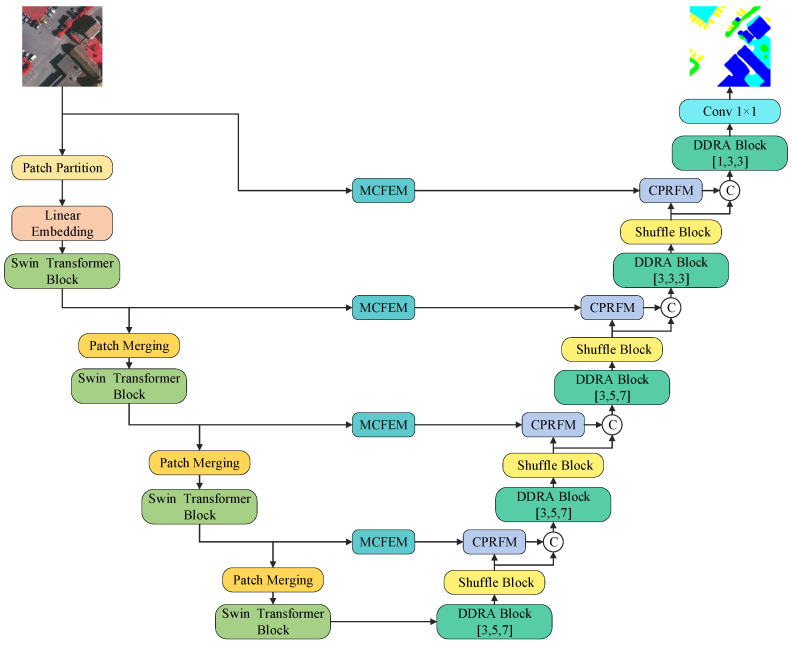
Overall architecture of MFPI-Net.

The overall architecture of our MFPI-Net is shown in Figure 1. The architecture is built under a U-shaped framework, where a pre-trained Swin Transformer backbone [29] network is selected as the encoder, which is composed of 4 Swin Transformer encoder blocks in cascade. The decoder is composed of a series of proposed DDRA Blocks and Shuffle Blocks, of which there are 5 DDRA Blocks and 4 Shuffle Blocks. In addition, MCFEM is designed to preserve low-level information with high resolution and combine the respective advantages of CNN and Transformer, taking into account the representation of spatial details and global semantics. CPRFM is responsible for the detailed fusion of features from different levels or paths, extracting multi-scale information through multi-branch convolution, and adopting the feature-enhanced residual multiplication and addition mechanism to achieve effective feature interaction and information integration, thereby improving the model’s ability to recognize objects in complex scenes.

Specifically, the Swin Transformer encoder first extracts multi-level global semantic features from the input image. Subsequently, these features are fed into the MCFEM to further enhance the expression of local details. In the DDRASD part, the features first pass through the DDRA Block to enhance the multi-scale context perception ability, and then the Shuffle Block improves the spatial resolution and channel interaction. The features enhanced by MCFEM are fused with the DDRASD output features through CPRFM to achieve the effective integration of multi-source information. After the above modules are repeatedly applied in multiple stages, the network finally outputs the semantic segmentation result. Figure 1 shows the connection relationship and overall structural framework between the above modules. The specific operations of each sub-module are introduced in detail in Section 3.1, Section 3.2, Section 3.3, Section 3.4, Section 3.5, and the corresponding calculation process is formally defined by Formulas (1)–(12).

### 3.1. Swin Transformer Encoder

**Figure 2:**
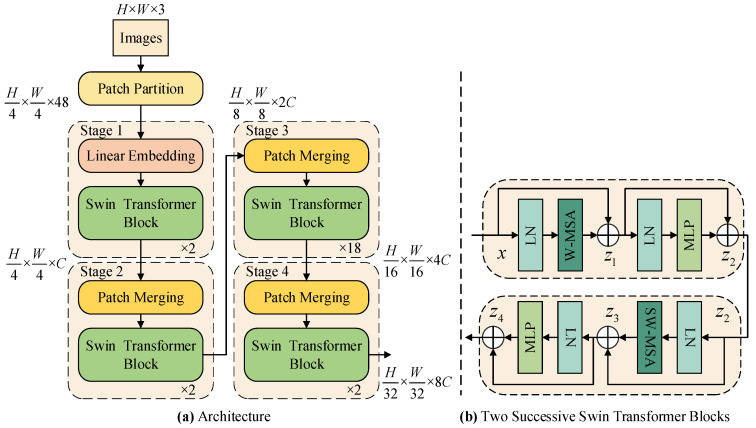
(**a**) The architecture of a Swin-B; (**b**) two successive Swin Transformer blocks.

The Swin Transformer encoder first labels the input image of size *H* × *W* × 3 through the Patch Embedding (Patch Partition and Linear Embedding) layer to generate a feature map: $\frac{H}{p}\;\times \;\frac{W}{p}\;\times \;C$. Then, the generated feature map passes through 4 Swin Transformer encoder blocks, and produces multi-level outputs of different sizes at each block: $\frac{H}{4}\;\times \;\frac{W}{4}\;\times \;C$, $\frac{H}{8}\;\times \;\frac{W}{8}\;\times \;2C$, $\frac{H}{16}\;\times \;\frac{W}{16}\;\times \;4C$, $\frac{H}{32}\;\times \;\frac{W}{32}\;\times \;8C$. Each Swin Transformer encoder block is followed by a Patch Merging layer, which reduces the spatial dimension to half of the original before passing the feature to the next deeper Swin Transformer encoder block. Swin Transformer provides multiple scale versions, mainly including Swin-Tiny (Swin-T), Swin-Small (Swin-S), and Swin-Base (Swin-B), which balance model complexity and performance by adjusting the depth, width (number of channels), and window size of the network. The Swin-B architecture used in this paper is shown in Figure 2. In Figure 2b, W-MSA and SW-MSA are the window-based multi-head self-attention mechanism and the shifted window multi-head self-attention mechanism, respectively. Other symbols are explained in Formulas (2)–(5).

(1) Patch Embedding: The Patch Embedding layer is responsible for converting the input image into a feature sequence suitable for Transformer processing, which includes two key steps: Patch Partition and Linear Embedding. First, Patch Partition divides the original RGB image into non-overlapping 4 × 4 pixel blocks (patches). Each block is flattened into a vector of length 4 × 4 × 3 = 48, which is equivalent to splitting the image into $\frac{H}{4}\;\times \;\frac{W}{4}$ patch tokens. Next, Linear Embedding projects each patch vector to the specified dimension through a fully connected layer. For example, the embedding dimension of Stage 1 in Swin-B is 128, forming a feature matrix of dimension $\frac{H}{4}\;\times \;\frac{W}{4}\;\times \;128$. This step is similar to “breaking” the image into feature blocks and giving each block a semantic representation suitable for deep network processing.
(2) Swin Transformer encoder block: The Swin Transformer encoder block is the core component for modeling image context information. By introducing a shift window strategy, SW-MSA enables information interaction between different windows, thereby enhancing the expressiveness of the model while maintaining a low computational cost. Each encoder block consists of a W-MSA or SW-MSA module and a multi-layer feedforward neural network (MLP), combined with layer normalization and residual connections, ensuring the stability of deep network training and excellent performance.

In the first encoder block, the input feature *x* is first normalized by LayerNorm (LN), and then the input is divided into several local windows of size *M* × *M*, each window containing *M*^2^ tokens. The attention calculation complexity of the traditional Transformer is $O(N^{2}\cdot d)$, that is, each token interacts with all other tokens, where *N* is the total number of tokens, and *d* is the feature dimension of each token. The Swin Transformer limits the attention to the local window, and only needs to calculate the attention between *M*^2^ tokens in each window, with a total complexity of $O(M^{2}\cdot N\cdot d)$, which greatly reduces the computational burden under high-resolution images. In each window, the input feature is first linearly projected to obtain the query *Q*, key *K*, and value *V*. In each attention head, local self-attention calculation is performed, and the formula [29] is as follows:

(1) $$\mathrm{Attention}(Q,K,V)=\mathrm{softmax}\left(\frac{QK^{T}}{\sqrt{d/h}}+B\right)V$$

where *h* is the number of attention heads, *T* represents transposition, and *B* is the relative position bias matrix, which is used to encode the spatial position of the local patch. The processed result is added to the original input to obtain the first-layer residual connection output *z*_1_. Subsequently, *z*_1_ is processed by LN and MLP, and its output is added to *z*_1_ to obtain the second-layer residual connection output *z*_2_. In order to enhance the information interaction between different windows, the second encoder block adopts SW-MSA to achieve cross-combination of originally separated areas by spatially shifting the windows. After normalization, attention, and MLP modules, outputs *z*_3_ and *z*_4_ are obtained, respectively.

(2) $$z_{1}=\mathrm{W-MSA}(\mathrm{LN}(x))+x$$

(3) $$z_{2}=\mathrm{MLP}(\mathrm{LN}(z_{1}))+z_{1}$$

(4) $$z_{3}=\mathrm{SW-MSA}(\mathrm{LN}(z_{2}))+z_{2}$$

(5) $$z_{4}=\mathrm{MLP}(\mathrm{LN}(z_{3}))+z_{3}$$

Through the continuous stacking of Swin Transformer encoder blocks, the model can gradually expand the receptive field while effectively controlling the computational complexity, achieving modeling from local details to global semantics.

(3) Patch Merging: The Patch Merging layer is used to reduce the spatial resolution of feature maps between layers and increase the channel dimension to achieve hierarchical modeling. Specifically, it first splices adjacent 2 × 2 patches to form a new patch. If the input feature map size is *H* × *W* and the number of channels is *C*, each 2 × 2 area is flattened into a vector of length 4*C* and reduced to 2*C* dimensions through linear mapping to achieve spatial downsampling and channel expansion. This operation retains key information while compressing the amount of computation, which helps capture image semantics at different scales.

### 3.2. Diverse Dilation Rates Attention Shuffle Decoder

**Figure 3:**
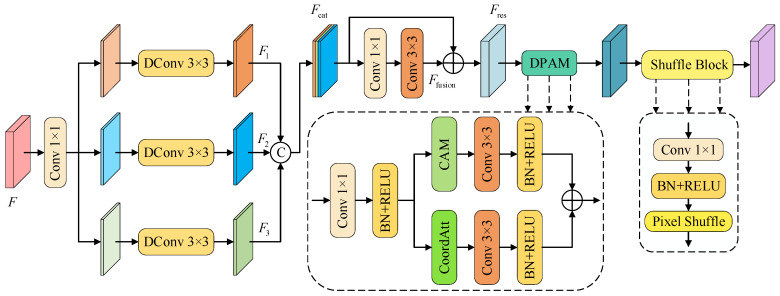
DDRASD structure diagram.

Although fine local features are crucial for the detection and segmentation of small objects, the introduction of global context information cannot be ignored in the decoding stage. To this end, we designed a DDRASD, which combines multi-scale modeling capabilities with efficient upsampling strategies to make better use of the multi-scale feature maps extracted by the encoder and achieve the accurate recovery of semantic information. The decoder consists of the DDRA Block and the Shuffle Block. Among them, the DDRA Block applies convolution operations with diverse dilation rates in parallel to effectively expand the receptive field and obtain context information from multiple scales; at the same time, it integrates channel attention and coordinate attention mechanisms to enhance the response of key areas in both spatial and channel dimensions, so that the model can more accurately locate and segment objects with significant size differences. In order to avoid the checkerboard effect introduced by conventional upsampling operations (such as deconvolution), we introduced Shuffle Block in the decoder to achieve efficient upsampling by rearranging pixels, which not only avoids the appearance of the checkerboard effect, but also improves the spatial resolution of the feature map without introducing additional parameters. The network structure of DDRASD is shown in Figure 3.

(1) DDRA Block: The DDRA Block is a structural reconstruction module designed for the decoder stage. It integrates multi-scale feature analysis, residual information guidance, and dual-path attention enhancement. The goal is to strengthen semantic understanding and structural perception, enhance the response strength of target regions, and achieve accurate restoration and detailed reconstruction in complex scenes. The module adopts a strategy that combines convolutional path extraction with diverse dilation rates, multi-level feature fusion, and attention-guided optimization. A total of five DDRA Blocks are deployed in the decoder. These modules construct appropriate receptive field structures by setting different dilation rates, thereby enabling the fine-grained modeling of features at different semantic levels. The first three modules employ convolutional paths with diverse dilation rates, using receptive field configurations of [3,5,7], and adopt a progressively increasing dilation strategy to capture multi-scale contextual information and enhance the semantic representation of deep features. The fourth module uses a uniform dilation configuration of [3] to form a standard receptive field structure, focusing on extracting local details from shallow features. The fifth module adopts a receptive field configuration of [1,3] to effectively integrate deep semantic and shallow spatial information, thereby improving segmentation accuracy and the structural restoration of small-object boundaries.

In each DDRA Block, the input feature $F\in R^{C\times H\times W}$ is first adjusted through 1 × 1 convolution to adjust the number of channels, and then divided into three groups of sub-channels. If the number of input channels cannot be divided by 3, the remainder is distributed to the last two branches in reverse order starting from the last branch. Then, the three groups of sub-channels are input into three 3 × 3 convolution paths with different dilation rates for processing. The effective receptive field of each path is determined by the following formula:

(6) $$r_{i}=1+(k-1)\cdot d_{i},i\in \{1,2,3\}$$

where *k* is 3, and *d_i_* is the dilation rate set for each branch. The outputs *F*_1_, *F*_2_, and *F*_3_ of the three branches are concatenated in the channel dimension to form a fusion feature *F*_cat_, which is then reduced in dimension by 1 × 1 convolution and enhanced with 3 × 3 convolution to obtain a unified fusion representation *F*_fusion_. In order to achieve effective feature compensation and improve the stability of the training process, the residual connection mechanism is introduced into the module, and a learnable weight parameter $\alpha \in \left[0,1\right]$ is used for weighted combination. The initial value of *α* is 0.5, indicating that the weights of new and old features are initially equal; during the training process, the network is allowed to autonomously adjust the fusion ratio according to different training stages, thereby dynamically balancing the information flow of new and old features. This design effectively alleviates the problem of gradient imbalance that may be caused by fixed fusion weights, and promotes the stability and convergence performance of model training.

(7) $$F_{\mathrm{fusion}}={\mathrm{Conv}}_{3\times 3}({\mathrm{Conv}}_{1\times 1}([F_{1};F_{2};F_{3}]))$$

(8) $$F_{\mathrm{res}}=\alpha \cdot F_{\mathrm{fusion}}+(1-\alpha )\cdot F_{\mathrm{cat}}$$

Finally, in order to enhance the model’s perception of channel semantics and spatial position information, the residual fusion feature *F*_res_ is sent to the Dual-Path Attention Module (DPAM), which consists of the Channel Attention Module (CAM) [43,44] and the coordinate attention (CoordAtt) mechanism. Both of them take into account the global semantic modeling of the channel dimension and the local structure perception of the spatial dimension in parallel, so that the model can accurately perceive the fine-grained spatial layout while maintaining global consistency, significantly improving the quality of structure restoration and the accuracy of small target recognition.

(2) Shuffle Block: The Shuffle Block uses the Pixel Shuffle mechanism to achieve 2× spatial upsampling. First, a 1 × 1 convolution is used to expand the number of channels of the input feature to 4 times the number of target channels to meet the channel reconstruction requirements of Pixel Shuffle. Subsequently, Batch Normalization and ReLU activation functions are introduced to standardize and nonlinearly map the convolution output to enhance the feature expression capability. Finally, the Pixel Shuffle operation is used to effectively rearrange the channel dimension to the spatial dimension. The Shuffle Block ultimately achieves a 2× expansion of the feature map spatial size and a compression of the number of channels to half of the original, significantly improving the spatial resolution of the feature map and avoiding the checkerboard artifacts that are easily generated by traditional deconvolution methods. This module does not require the introduction of additional parameters, has high computational efficiency, and helps to preserve structural information and improve upsampling quality. In the network structure, a total of four Shuffle Blocks are designed, which are placed after the first four DDRA Blocks, gradually restore the spatial resolution of the feature map, and achieve multi-level feature detail enhancement.

### 3.3. Multi-Scale Convolutional Feature Enhancement Module

**Figure 4:**
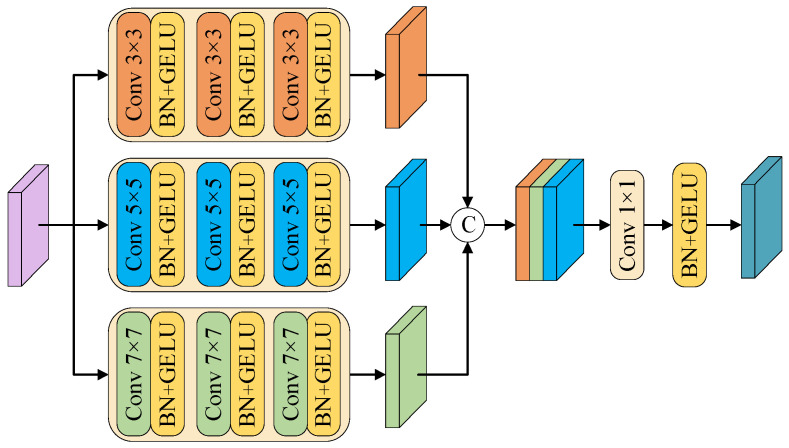
MCFEM structure diagram.

In order to enhance the model’s perception of multi-scale targets and complex structures in urban remote sensing images, this paper proposes a multi-scale convolutional feature enhancement module and integrates it into the jump connections of each layer output in the input stage and encoding stage of the network, thereby introducing richer local spatial structures and multi-scale detail information while maintaining the global modeling advantages of Swin Transformer, and enhancing the model’s ability to express the target morphology and scale changes in complex remote sensing scenes. As shown in Figure 4, the MCFEM consists of three parallel convolution branches, using 3 × 3, 5 × 5 and 7 × 7 kernel sizes to perceive fine-grained, medium-scale and large-scale spatial context information. Each branch consists of a three-layer convolution structure, which gradually extracts local spatial features and multi-scale context information through continuous convolution operations and enhances the structural expression ability. All convolutional layers are equipped with Batch Normalization and GELU activation functions to improve feature stability and nonlinear expression ability. The three-way output features are concatenated in the channel dimension and compressed by 1 × 1 convolution fusion to form a unified multi-scale feature representation.

The MCFEM forms a collaborative modeling mechanism with the Swin Transformer backbone at two key levels: First, in the input stage, the MCFEM extracts the underlying multi-scale structural features from the original image as input, and transmits them to the decoder through jump connections, effectively supplementing the inadequacy of Swin Transformer in modeling low-level information such as local texture and edge details. Second, in the encoding stage, the MCFEM is integrated into the jump connection path of the output of the first three layers of Swin Transformer. The sequence features generated by Swin Transformer are first restored to a two-dimensional spatial structure, and then semantically enhanced through multi-scale convolution. The obtained features are used to assist the decoder to more accurately restore the target structure and boundary information. Through this deep collaboration in the two stages of input and encoding, the MCFEM gives full play to the strengths of convolutional networks in modeling local spatial structures and fine-grained details, forming a complementary relationship with the Swin Transformer’s global modeling capabilities, thereby improving the model’s structural perception and scale generalization capabilities for different types of targets in urban remote sensing images.

### 3.4. Cross-Path Residual Fusion Module

**Figure 5:**
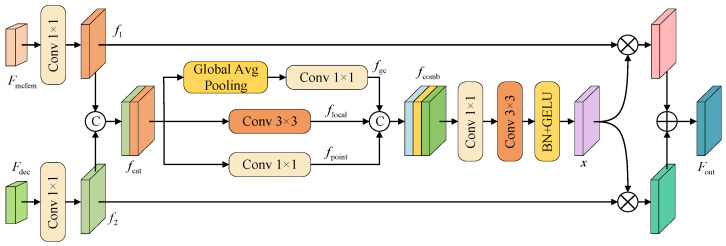
CPRFM structure diagram.

In order to achieve the effective fusion of multi-scale semantics and detail features and enhance the model’s ability to identify fine-grained targets and similar easily confused categories in complex scenarios, we designed the CPRFM, as shown in Figure 5. This module is deployed on the jump connection path in the decoder structure and is dedicated to integrating feature information from different semantic levels. On the one hand, it receives features from the shallow layer of the encoder. After being processed by MCFEM, this branch carries rich local texture and structural details; on the other hand, it receives high-level semantic features from the trunk decoding path and has strong global semantic understanding capabilities. The CPRFM fuses shallow detail features with deep semantic features to enhance the complementarity and discrimination of features.

In order to fully integrate these two types of features, the CPRFM first performs 1 × 1 convolution on the two input features *F*_mcfem_ and *F*_dec_ to unify the channel dimension, and aligns the spatial dimensions through bilinear interpolation to obtain *f*_1_ and *f*_2_. They are spliced along the channel dimension to form a preliminary fusion feature:

(9) $$f_{\mathrm{cat}}=\mathrm{Concat}(f_{1},f_{2})$$

In order to simultaneously model global semantics and local detail information, the CPRFM designs three parallel paths to extract features from *f*_cat_. Among them, the global context branch compresses the spliced features into a global description vector through global average pooling to capture the overall semantic information of the image. Subsequently, 1 × 1 convolution is used for channel transformation, and the global features are upsampled to the original feature size through bilinear interpolation to achieve spatial alignment. The local detail branch uses 3 × 3 convolution to focus on local details such as edges and textures, and effectively supplements the detail information that is difficult to cover by the global branch through a medium receptive field, thereby enhancing the expression of target boundaries and fine-grained areas. The point convolution branch strengthens the expression of inter-channel features through 1 × 1 convolution. The three paths collaboratively model global semantics, local structure, and channel relationships, and finally concatenate the three features to form a fused feature:

*f*_comb_ = Contanct(*f*_gc_, *f*_local_, *f*_point_) (10)

After the fused feature is subjected to 1 × 1 convolution and 3 × 3 convolution, Batch Normalization and GELU activation function, an enhanced feature *x* is generated. To further enhance the interaction and coupling between features from different sources, CPRFM introduces a residual multiplication and addition mechanism: feature *x* is multiplied element by element with *f*_1_ and *f*_2_, respectively, and then added to obtain the final fusion result; the calculation formula is shown in Equation (11).

*F*_out_ = *f*_1_·*x* + *f*_2_·*x* (11)

The CPRFM achieves the organic unity of semantic enhancement and detail retention while ensuring computational efficiency by guiding the deep interaction between different path features, effectively improving the model’s recognition ability for fine-grained targets and easily confused categories.

### 3.5. Loss Function

In the task of semantic segmentation of remote sensing images, the goal is to perform semantic classification on each pixel in the high-resolution image to achieve the accurate identification of the object category. To this end, the model needs to learn the mapping relationship between the input image and the corresponding label at the pixel level. During the training process, the cross-entropy loss function is often used as the optimization target to measure the difference between the category probability distribution predicted by the model and the true label. This paper uses the cross-entropy loss function to supervise the network, and its formula is as follows:

(12) $$L_{CE}=-\sum_{i=1}^{n}y_{i}\log({\hat{y}}_{i})$$

where *n* is the number of classification categories, *y_i_* is the true value, and ${\hat{y}}_{i}$ is the softmax probability of the *i*-th class.

## 4. Experimental Settings and Datasets

### 4.1. Datasets

We evaluate the proposed model on two urban remote sensing image datasets, ISPRS Vaihingen and ISPRS Potsdam, as shown below.

(1) ISPRS Vaihingen: The ISPRS Vaihingen dataset is a standard remote sensing image semantic segmentation benchmark dataset released by the International Society for Photogrammetry and Remote Sensing (ISPRS). It is widely used in pixel-level object classification research in urban scenes. The dataset contains 33 high-resolution images, including five categories: impervious surface, building, low vegetation, tree, and car, as well as a cluttered background category. Due to the high resolution of the original images, in order to adapt to the video memory and computing limitations during model training, we use a sliding window strategy to crop them into small tiles of 512 × 512 pixels. On this basis, in order to enhance the diversity of image directions and improve the model’s learning ability and robustness for target features in different directions, the image is randomly flipped horizontally or vertically with a probability of 0.5. In addition, in order to enhance the model’s adaptability under various lighting conditions, a photometric distortion operation is introduced, including random adjustments to the image’s brightness, contrast, saturation, and hue, thereby simulating a complex lighting environment. The dataset is randomly divided into a training set, a validation set, and a test set in a ratio of 8:1:1, and finally 6820 training images, 852 validation images, and 852 test images are obtained.
(2) ISPRS Potsdam: The ISPRS Potsdam dataset is another standard benchmark dataset released by ISPRS and widely used in remote sensing image semantic segmentation tasks. It is mainly used to evaluate the pixel-level classification performance of models in high-resolution urban remote sensing scenes. This dataset contains 38 high-resolution images, and the categories are consistent with the Vaihingen dataset, including five categories: impervious surface, building, low vegetation, tree, car, and cluttered background. For the processing of this dataset, we also used the sliding window strategy to crop the original image into small tiles of 512 × 512 pixels, and used the same data augmentation strategy to improve the robustness and generalization ability of the model. The dataset was randomly divided into training set, validation set and test set in a ratio of 8:1:1, and finally 12,696 training images, 1587 validation images, and 1587 test images were obtained.

### 4.2. Experimental Environment and Parameter Configuration

All our experiments were conducted in Python 3.8, under the PyTorch 1.13.0 framework, and on an NVIDIA 3090 GPU. The AdamW [45] optimizer was used for model training, with an initial learning rate of 6 × 10^−5^, a weight decay rate of 0.01, and a beta parameter of (0.9, 0.999). The learning rate adjustment strategy used a Poly strategy based on exponential decay, and the entire training process lasted for 160,000 iterations. The batch size in the training phase is set to 2, and the batch size in the testing phase is set to 1.

### 4.3. Evaluation Metrics

We use intersection over union (IoU), mean intersection over union (mIoU), overall accuracy (OA), and mean F1 score (mF1) as evaluation indicators of model performance. It should be noted that, in the process of network performance evaluation, the cluttered background class does not participate in the calculation of evaluation indicators. These indicators are calculated based on four key parameters in the prediction results: true positive (TP), true negative (TN), false positive (FP), and false negative (FN). Based on these four values, for a single category $k\in \left[1,2,\ldots ,K\right]$ (where *K* represents the total number of categories), its IoU, accuracy (Acc), and F1 score are calculated as follows:

(13) $${\mathrm{IoU}}_{k}=\frac{TP_{k}}{TP_{k}+FP_{k}+FN_{k}}$$

(14) $${\mathrm{Acc}}_{k}=\frac{TP_{k}+TN_{k}}{TP_{k}+FP_{k}+TN_{k}+FN_{k}}$$

(15) $${F1}_{k}=\frac{2TP_{k}}{2TP_{k}+FP_{k}+FN_{k}}$$

We further calculate the average performance indicators on all categories. The calculation methods of mIoU, OA, and mF1 are as follows:

(16) $$\mathrm{mIoU}=\frac{1}{K}\sum_{k=1}^{K}{\mathrm{IoU}}_{k}$$

(17) $$\mathrm{OA}=\frac{1}{K}\sum_{k=1}^{K}{\mathrm{Acc}}_{k}$$

(18) $$\mathrm{mF}1=\frac{1}{K}\sum_{k=1}^{K}{F1}_{k}$$

## 5. Experimental Results and Analysis

### 5.1. Comparative Experimental Design and Results Analysis

In order to evaluate the effectiveness of the proposed model MFPI-Net, we conducted multiple experiments on two urban remote sensing image datasets. Each dataset saved the model with the best performance on the validation set during the training process, and selected seven current mainstream semantic segmentation models for comparative analysis, including UNet, DeepLabV3+, EAS-CNN, TransUNet, DC-Swin, SSNet [46], and ConvNeXt-Mask2Former [47], covering a variety of models based on CNN, Transformer, and their hybrid structures.

#### 5.1.1. Comparative Analysis on the Vaihingen Dataset

**Figure 6:**
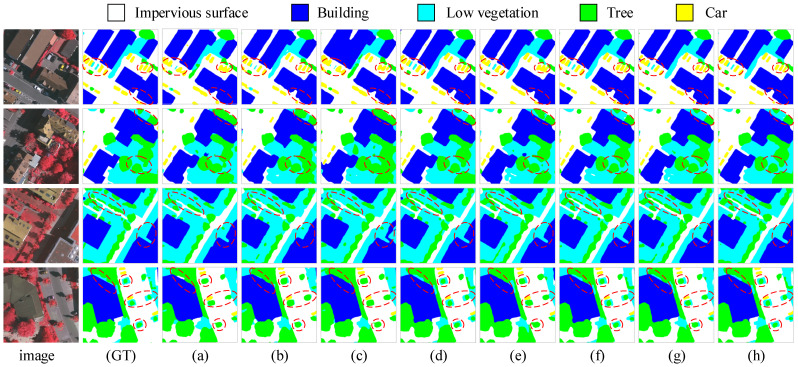
Segmentation results of different models on the Vaihingen dataset. The first column image is the original remote sensing image, the second column (GT) is the corresponding true label, and (**a**–**h**) are the segmentation results of UNet, DeepLabV3+, TransUNet, DC-Swin, EAS-CNN, SSNet, ConvNext-Mask2Former, and MFPI-Net, respectively.

We compared the performance of the proposed MFPI-Net with the current mainstream semantic segmentation models on the Vaihingen dataset, and the results are shown in Table 1. As can be seen from the table, MFPI-Net outperforms other models in overall performance. Specifically, MFPI-Net’s mIoU reaches 82.57%, which is 0.56 percentage points higher than the second-best model DC-Swin (82.01%); mF1 reaches 90.28%, which is 0.35 percentage points higher than DC-Swin (89.93%); OA reaches 91.23%, which is 0.06 percentage points higher than DC-Swin (91.17%). In terms of segmentation accuracy of each category, MFPI-Net performs best in the IoU indicators of impervious surfaces (87.76%), low vegetation (72.71%), trees (80.28%), and vehicles (78.81%), showing good adaptability and robustness to different ground object categories. Although ConvNext-Mask2Former’s IoU reaches 93.36% in the building category, which is slightly better than our method, MFPI-Net has achieved comprehensive surpassing in the overall evaluation indicators with more balanced performance in various categories. Figure 6 shows the comparison of segmentation effects of different models on the Vaihingen dataset. It can be seen that the prediction results of MFPI-Net are highly consistent with the true labels, showing excellent segmentation performance. The area marked by the red dotted line in the figure further highlights the significant advantages of the model in boundary characterization, small target detail restoration, and similar category recognition. Compared with other models, MFPI-Net not only performs better in overall semantic consistency, but also can more accurately capture fine-grained information in complex scenes, fully verifying its strong segmentation ability and semantic understanding level in complex urban scenes.

#### 5.1.2. Comparative Analysis on the Potsdam Dataset

**Figure 7:**
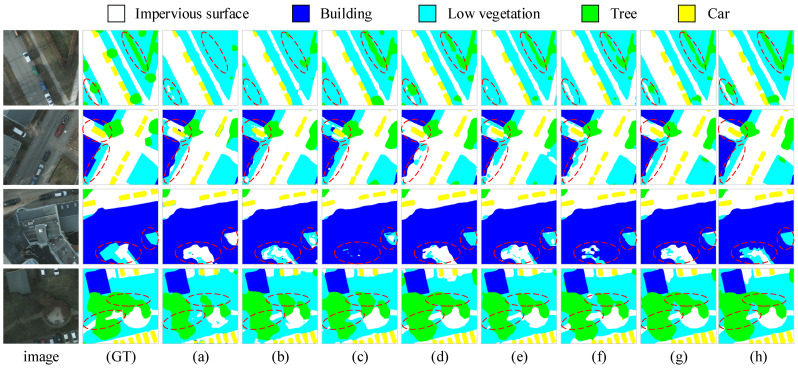
Segmentation results of different models on the Potsdam dataset. The first column image is the original remote sensing image, the second column (GT) is the corresponding true label, (**a**–**h**) are the segmentation results of UNet, DeepLabV3+, TransUNet, DC-Swin, EAS-CNN, SSNet, ConvNext-Mask2Former, and MFPI-Net, respectively.

We compared the performance of the proposed MFPI-Net with the current mainstream semantic segmentation model on the Potsdam dataset. The specific results are shown in Table 2. It can be clearly seen that MFPI-Net has a significant advantage in overall performance. Among them, the model achieved 88.49% in mIoU, which is 1.3 percentage points higher than the second-performing DC-Swin (87.19%); it reached 93.77% in the mF1 index, which is about 0.76 percentage points higher than DC-Swin (93.01%); OA is 93.64%, which is also 0.59 percentage points higher than DC-Swin’s 93.05%. From the perspective of segmentation performance in various categories, MFPI-Net also shows strong performance, with the highest IoU values in the five categories of impervious surfaces (90.92%), buildings (96.03%), low vegetation (80.93%), trees (80.55%), and vehicles (94.02%), indicating that it has good generalization ability and stability when dealing with different types of urban elements. In addition, from the visual comparison results shown in Figure 7, it can be seen that MFPI-Net is highly close to the true label in terms of prediction accuracy, especially in the ability to distinguish similar categories and accurately identify small target details. Its segmentation results are better than the comparison model in terms of semantic coherence and edge detail processing, further verifying MFPI-Net’s excellent parsing ability and excellent semantic understanding level in complex urban scenes.

### 5.2. Ablation Experiment Design and Results Analysis

**Figure 8:**
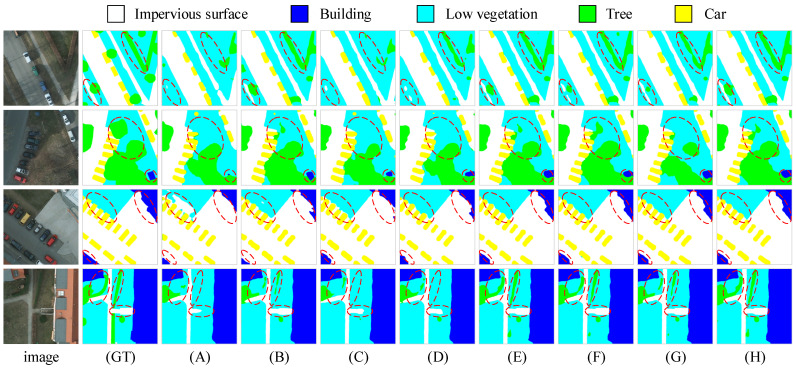
Visualization results of ablation experiment on Potsdam dataset. The first column image is the original remote sensing image, the second column (GT) is the corresponding true label, and (**A**–**H**) are the segmentation results of baseline, baseline+DDRASD, baseline+MCFEM, baseline+CPRFM, baseline+DDRASD+MCFEM, baseline+DDRASD+CPRFM, baseline+MCFEM+CPRFM, and MFPI-Net, respectively.

To verify the effectiveness of each key module in MFPI-Net, we conducted a systematic ablation experiment on the Potsdam dataset. The results are shown in Table 3. In the table, “×” indicates that the module is not included in the model, and “√” indicates that the module is included in the model. The baseline model uses Swin-B as the backbone network to build an encoder–decoder structure. On this basis, three core modules are gradually introduced, i.e., DDRASD, MCFEM, and CPRFM, to evaluate the impact of single modules and combined modules on model performance. The visualization results of the ablation experiment are shown in Figure 8.

From the experimental results, the baseline model has mIoU, mF1, and OA of 81.66%, 89.71%, and 89.99%, respectively, without adding any modules. When only the DDRASD is introduced, mIoU is significantly improved to 85.51%, and mF1 and OA are also improved to 92.00% and 92.01%, respectively, indicating that the decoder structure plays a core role in feature decoding and context modeling, especially in multi-scale perception and edge detail preservation, which is a key factor in improving overall performance. When the MCFEM is introduced alone, the model mIoU is improved to 83.44%, mF1 to 90.69%, and OA to 90.81%, indicating that this module has a good complementary role in fusing local spatial information with global semantic information, and effectively enhances the feature expression ability at multiple scales. When the CPRFM is added alone, mIoU rises slightly to 82.94%, and mF1 and OA are 90.44% and 90.46%, respectively. Although the individual effect is not as obvious as that of the DDRASD or the MCFEM, the CPRFM shows good synergistic gains when used in conjunction with other modules.

When two modules are used in combination, the performance is further improved. For example, when the DDRASD and the MCFEM (without the CPRFM) are introduced, the mIoU reaches 87.88%, and when the DDRASD is combined with the CPRFM, an mIoU of 87.76% is also achieved, both of which are significantly better than the effect of any module acting alone. This shows that the DDRASD can make more full use of multi-source features when working with the MCFEM or the CPRFM to achieve more accurate semantic reconstruction. Finally, when all three modules are fused, the model performance reaches the best, with mIoU of 88.49%, mF1 of 93.77%, and OA of 93.64%, which is better than other combinations. This shows that the modules complement each other in terms of functional positioning. Through multi-scale modeling, attention enhancement, feature fusion, and other means, the recognition and segmentation capabilities of the model in complex urban scenes are effectively improved, verifying the rationality of MFPI-Net design and module synergy.

### 5.3. The Impact of Different Scale Swin Transformer Backbones on Model Performance

To explore the impact of the backbone network scale on the performance of MFPI-Net in urban remote sensing semantic segmentation tasks, we kept the core modules (DDRASD, MCFEM, CPRFM) of the model unchanged and used three typical scale versions of Swin Transformer, i.e., Tiny (Swin-T), Small (Swin-S), and Base (Swin-B), as the backbone network to conduct experiments on the Vaihingen and Potsdam datasets. The network depth and width of these three versions are different. The specific parameters are shown in Table 4, and the experimental results are shown in Table 5.

Experimental results show that the scale of Swin Transformer variants has a significant impact on the performance of MFPI-Net. On the Vaihingen dataset, when Swin-B is used as the backbone network, the model achieves optimal performance in all evaluation indicators, with mIoU reaching 82.57%, mF1 reaching 90.28%, and OA reaching 91.23%; on the Potsdam dataset, when Swin-B is also used as the backbone network, the model performance achieves the best performance, with mIoU, mF1, and OA reaching 88.49%, 93.77%, and 93.64%, respectively. This shows that, as Swin Transformer is upgraded from Tiny to Base, the increase in network depth and width enhances the model’s ability to express the features of complex scenes in urban remote sensing images, thereby promoting the accuracy of MFPI-Net in the semantic segmentation task of urban remote sensing images.

### 5.4. Training Process and Confusion Matrix Visualization Analysis

#### 5.4.1. Visual Analysis of the Training Process

**Figure 9:**
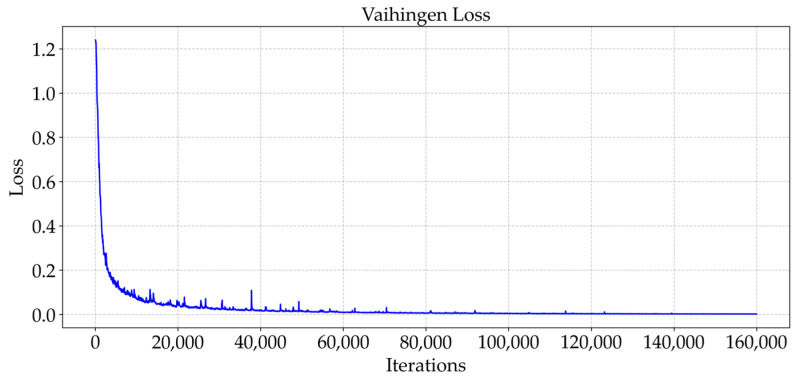
Loss change curve of the Vaihingen dataset.

**Figure 10:**
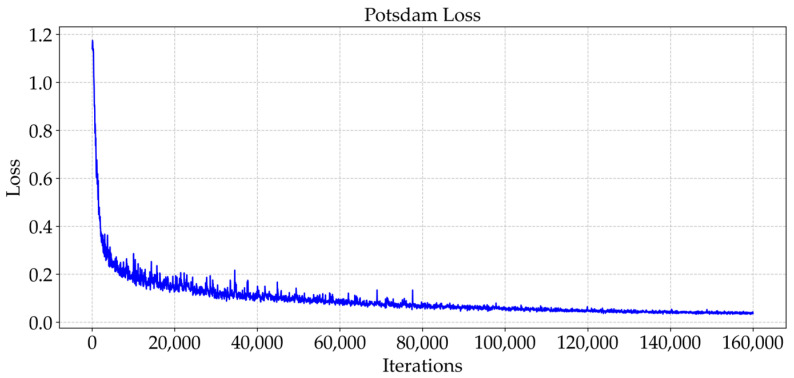
Loss change curve of the Potsdam dataset.

**Figure 11:**
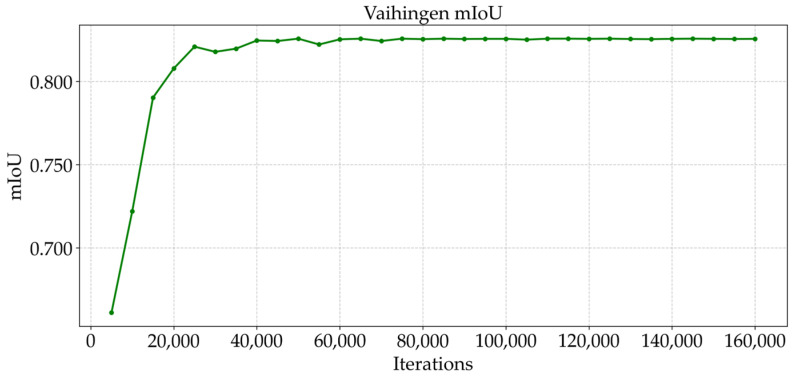
mIoU change curve of the Vaihingen dataset.

**Figure 12:**
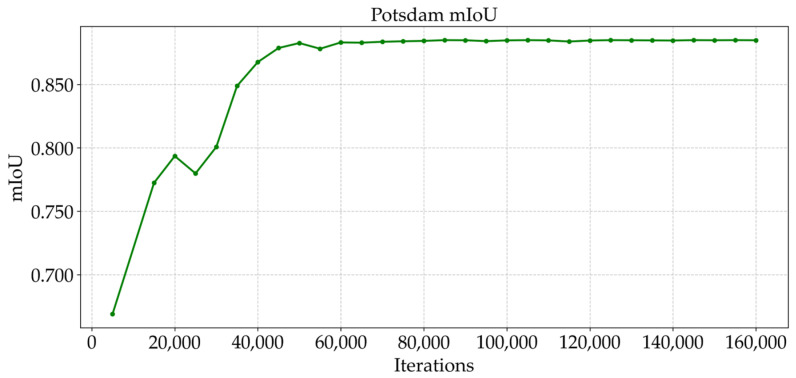
mIoU change curve of the Potsdam dataset.

As shown in Figure 9 and Figure 10, the trend of the cross-entropy loss value with the number of training iterations during the training of MFPI-Net on the two datasets is shown. Figure 11 and Figure 12 show the change in the model’s mIoU on the validation set with the training process. It can be observed that, as the training progresses, the cross-entropy loss continues to decrease and gradually stabilizes, while the mIoU on the validation set steadily increases and eventually reaches a high and stable level. These results show that the proposed MFPI-Net has good loss function convergence during the training process, and the mIoU on the validation set steadily improves and eventually converges, showing the stability and reliability of the model training.

#### 5.4.2. Confusion Matrix Visualization Analysis

**Figure 13:**
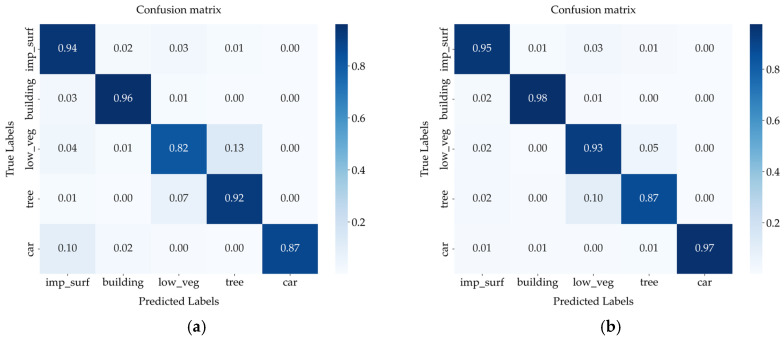
Confusion matrix of MFPI-Net under two datasets. (**a**) Confusion matrix for the Vaihingen dataset. (**b**) Confusion matrix for the Potsdam dataset.

On the two datasets, we generate a visualization of the confusion matrix for the proposed MFPI-Net, as shown in Figure 13a,b; these figures show the classification performance of the model on the Vaihingen and Potsdam datasets, respectively. imp_surf and low_veg in Figure 13 represent impervious surface and low vegetation, respectively.

From the overall performance of the confusion matrix, the model has good discrimination ability between different categories, and the values on the main diagonal are high, indicating that most pixels are accurately classified. Although there is some confusion between some categories, the overall classification accuracy is still high, indicating that the proposed method has strong discrimination ability and stable performance. In addition, both sets of confusion matrices show that the model still maintains good classification results when dealing with similar categories and small objects, further verifying the practicality of the model.

## 6. Conclusions

In view of the challenges of multi-scale distribution of objects, high similarity between categories, difficulty in identifying small targets, and easily missed detection in the semantic segmentation of urban remote sensing images, this paper proposes a semantic segmentation network MFPI-Net for urban remote sensing images based on multi-scale feature perception and interaction. The model adopts an encoder–decoder framework and integrates multiple structural innovations to effectively improve the accuracy and robustness of semantic segmentation of urban remote sensing images. Specifically, in the feature decoding stage, MFPI-Net designs the DDRASD composed of the DDRA Block and the Shuffle Block. The DDRA Block captures contextual information of different scales through multi-expansion rate convolution, and introduces channel and coordinate attention mechanisms to improve the model’s perception of multi-scale targets. The Shuffle Block improves the resolution of feature maps by rearranging pixels, avoiding the checkerboard artifacts in traditional deconvolution operations, and achieving fine restoration of boundary information. In the feature enhancement and fusion stage, the model introduces the MCFEM and the CPRFM. Among them, the MCFEM employs parallel multi-kernel convolution branches to enhance the CNN’s capability in local feature extraction, forming a complementary relationship with the Transformer’s long-range dependency modeling, and achieves the coordination of spatial details and semantic global information. The CPRFM integrates features of different scales and levels through multi-branch convolution paths, and adopts residual multiplication and addition fusion mechanism to enhance the complementarity and distinguishability between features, thereby improving the model’s ability to recognize similar categories and small targets in complex urban scenes. Experimental results show that MFPI-Net has achieved excellent performance in core evaluation indicators such as mIoU, mF1, and OA, demonstrating its effectiveness in the semantic segmentation task of urban remote sensing images. It should be pointed out that the current model has about 178.6M parameters, which results in high-precision performance but also places certain requirements on computing resources. Future work will focus on algorithm optimization to further improve the segmentation accuracy of the model. At the same time, we will focus on reducing the number of model parameters and building a lighter architecture to speed up model training.

## Figures and Tables

**Table 1:** Comparison results of different models on the Vaihingen dataset. Bold values denote the best performance.

Method	Class IoU%					mIoU %	mF1 %	OA %
	Impervious Surface	Building	Low Vegetation	Tree	Car			
UNet	79.49	90.59	69.78	77.42	72.38	77.93	87.42	88.46
DeepLabV3+	80.22	90.95	70.34	76.83	76.51	78.97	88.09	88.66
TransUNet	81.83	89.26	68.27	77.42	72.99	77.95	87.43	88.29
DC-Swin	87.60	93.26	72.59	80.27	76.31	82.01	89.93	91.17
EAS-CNN	87.51	92.94	71.22	79.63	72.70	80.80	89.15	90.83
SSNet	86.99	92.57	71.88	79.91	74.67	81.20	89.43	90.81
ConvNext-Mask2Former	87.41	**93.36**	71.86	79.68	75.31	81.53	89.62	90.95
MFPI-Net	**87.76**	93.29	**72.71**	**80.28**	**78.81**	**82.57**	**90.28**	**91.23**

**Table 2:** Comparison results of different networks on the Potsdam dataset. Bold values denote the best performance.

Method	Class IoU%					mIoU %	mF1 %	OA %
	Impervious Surface	Building	Low Vegetation	Tree	Car			
UNet	84.04	93.02	62.97	52.29	83.03	75.07	84.88	85.67
DeepLabV3+	86.38	92.08	73.35	72.37	82.79	81.39	89.55	90.11
TransUNet	85.29	87.22	71.64	74.18	85.24	80.71	89.18	89.24
DC-Swin	90.17	95.53	79.31	79.09	91.82	87.19	93.01	93.05
EAS-CNN	87.12	94.32	74.74	76.20	90.53	84.58	91.45	91.25
SSNet	88.03	94.01	76.18	74.17	88.10	84.10	91.17	91.43
ConvNext-Mask2Former	89.97	95.34	78.84	77.36	92.07	86.72	92.72	92.71
MFPI-Net	**90.92**	**96.03**	**80.93**	**80.55**	**94.02**	**88.49**	**93.77**	**93.64**

**Table 3:** Ablation experiment results on the Potsdam dataset. Bold values denote the best performance.

Model Name	Modules			mIoU %	mF1 %	OA %
	DDRASD	MCFEM	CPRFM			
A	×	×	×	81.66	89.71	89.99
B	√	×	×	85.51	92.00	92.01
C	×	√	×	83.44	90.69	90.81
D	×	×	√	82.94	90.44	90.46
E	√	√	×	87.88	93.41	93.35
F	√	×	√	87.76	93.34	93.22
G	×	√	√	85.43	91.87	92.04
H	√	√	√	**88.49**	**93.77**	**93.64**

**Table 4:** Parameter settings of Swin Transformer of different scales.

Network	Block Number	Channel Number	Heads
Swin-T	(2, 2, 6, 2)	96	(3, 6, 12, 24)
Swin-S	(2, 2, 18, 2)	96	(3, 6, 12, 24)
Swin-B	(2, 2, 18, 2)	128	(4, 8, 16, 32)

**Table 5:** The performance impact of different scales of Swin Transformer on the MFPI-Net model. Bold values denote the best performance.

Network	Vaihingen			Potsdam		
	mIoU%	mF1%	OA%	mIoU%	mF1%	OA%
Swin-T	79.73	88.58	89.08	85.51	92.01	91.89
Swin-S	81.98	89.92	90.83	87.88	93.41	93.29
Swin-B	**82.57**	**90.28**	**91.23**	**88.49**	**93.77**	**93.64**

## Data Availability

The original contributions presented in the study are included in the article, and further inquiries can be directed to the corresponding author.
